# Slow and Fast Neocortical Oscillations in the Senescence-Accelerated Mouse Model SAMP8

**DOI:** 10.3389/fnagi.2017.00141

**Published:** 2017-05-31

**Authors:** Patricia Castano-Prat, Maria Perez-Zabalza, Lorena Perez-Mendez, Rosa M. Escorihuela, Maria V. Sanchez-Vives

**Affiliations:** ^1^Systems Neuroscience, Institut d’Investigacions Biomèdiques August Pi i Sunyer (IDIBAPS)Barcelona, Spain; ^2^Departament de Psiquiatria i Medicina Legal, Institut de Neurociències, Universitat Autònoma de BarcelonaBarcelona, Spain; ^3^ICREABarcelona, Spain

**Keywords:** slow waves, slow oscillations, aging, gamma, cerebral cortex, Up states, Alzheimer, brain rhythms

## Abstract

The senescence-accelerated mouse prone 8 (SAMP8) model is characterized by accelerated, progressive cognitive decline as well as Alzheimer’s disease (AD)-like neurodegenerative changes, and resembles the etiology of multicausal, sporadic late-onset/age-related AD in humans. Our aim was to find whether these AD-like pathological features, together with the cognitive deficits present in the SAMP8 strain, are accompanied by disturbances in cortical network activity with respect to control mice (SAM resistance 1, SAMR1) and, if so, how the alterations in cortical activity progress with age. For this purpose, we characterized the extracellular spontaneous oscillatory activity in different regions of the cerebral cortex of SAMP8 and SAMR1 mice under ketamine anesthesia at 5 and 7 months of age. Under these conditions, slow oscillations and fast rhythms generated in the cortical network were recorded and different parameters of these oscillations were quantified and compared between SAMP8 and their control, SAMR1 mice. The average frequency of slow oscillations in SAMP8 mice was decreased with respect to the control mice at both studied ages. An elongation of the silent periods or Down states was behind the decreased slow oscillatory frequency while the duration of active or Up states remained stable. SAMP8 mice also presented increased cycle variability and reduced high frequency components during Down states. During Up states, the power peak in the gamma range was displaced towards lower frequencies in all the cortical areas of SAMP8 with respect to control mice suggesting that the spectral profile of SAMP8 animals is shifted towards lower frequencies. This shift is reminiscent to one of the principal hallmarks of electroencephalography (EEG) abnormalities in patients with Alzheimer’s disease, and adds evidence in support of the suitability of the SAMP8 mouse as a model of this disease. Although some of the differences between SAMP8 and control mice were emphasized with age, the evolution of the studied parameters as SAMR1 mice got older indicates that the SAMR1 phenotype tends to converge with that of SAMP8 animals. To our knowledge, this is the first systematic characterization of the cortical slow and fast rhythms in the SAMP8 strain and it provides useful insights about the cellular and synaptic mechanisms underlying the reported alterations.

## Introduction

Senescence accelerated mouse (SAM) strains are animal models of accelerated aging that occur naturally and spontnaneously, and were established through phenotypic selection instead of genetic engineering procedures (Takeda, 1999, 2009). Specifically, the SAM prone 8 (SAMP8) sub-strain presents accelerated, progressive cognitive decline as well as Alzheimer’s disease (AD)-like neurodegenerative changes with a low incidence of other aging phenotypes (Morley, 2002; Pallàs, 2012); conversely, SAM resistance 1 (SAMR1) mice present a normal aging pattern (Takeda, 1999). The etiology of the SAMP8 mouse model resembles the etiology of multicausal, sporadic late-onset/age-related AD in humans. For example, a partial correlation with several cell cycle markers has been described between human AD brains and SAMP8 mouse brains (Casadesús et al., 2012), and it has been suggested that SAMP8 mice may closely represent the complexity of the disease given the multifactorial nature of AD (Pallas et al., 2008; Morley et al., 2012; Cheng et al., 2014). Neuropathological AD-like hallmarks described in SAMP8 mice include impaired synaptic plasticity in hippocampal newborn slices (Yang et al., 2004) and hippocampal astrogliosis (Yagi et al., 1989), Aβ_40_-positive vessels (del Valle et al., 2011) and phosphorylated Tau (Tajes et al., 2008) at 3–4 months old. Neuronal loss, microgliosis and neurofibrillary tangles have been found in the neocortex at 5–6 months of age (Sureda et al., 2006), together with a reduction in dendritic spine density in pyramidal neurons of the CA1 hippocampal region, related to memory deficits in behaving SAMP8 mice (del Valle et al., 2012). Impairments in cognitive behavioral tasks suggestive of altered hippocampal function (Chen et al., 2004; Orejana et al., 2012), as well as disrupted amygdala-prefrontal cortex communication (Ohta et al., 2001) and cortex-dependent declarative memory (López-Ramos et al., 2012), have also been reported at 6 months old. The conjunction of neuropathological, morphological and connectivity alterations present in the SAMP8 strain are likely to result in alterations in functional cortical network activity patterns, alterations that have not yet been described in this mouse model.

There are different potential approaches for the identification of such altered patterns. In this study we opted for a meso-scale study of the cortical network function that uses as a paradigm the spontaneous activity during slow wave sleep or deep anesthesia, namely the slow oscillation (SO). The analysis of SO provides information about different parameters of the cortical function (e.g., excitability, wave propagation, power in different frequency bands) and has been used before for the characterization of different mouse models of disease (Gibson et al., 2008; Busche et al., 2015; Ruiz-Mejias et al., 2016). Cortical SO consist in a rhythmic pattern (≤1 Hz) with alternating periods of neuronal firing and synaptic activity, or Up states, interspersed with rather silent, inactive periods, or Down states (Steriade et al., 1993). Persistent activity during Up states is maintained by the recurrency within the network, and its properties and frequency content are often comparable to those occurring during cortical processing in the awake state (Steriade et al., 1996b; Steriade and Timofeev, 2003; MacLean et al., 2005; Destexhe et al., 2007; Luczak et al., 2007; Compte et al., 2008). SO have been described in different species (Haider et al., 2006; Sakata and Harris, 2009; Chauvette et al., 2010) including humans (Massimini et al., 2004; Csercsa et al., 2010), using both *in vitro* (Sanchez-Vives and McCormick, 2000; Rigas and Castro-Alamancos, 2007) and *in vivo* (Fellin et al., 2009; Ruiz-Mejias et al., 2011) conditions. Therefore, the SO constitutes a well-preserved and characterized network phenomenon that could be indicative of underlying alterations at the cellular and synaptic levels.

The aim of this study was to determine whether SAMP8 mice present disturbances in spontaneous cortical network activity when compared with the normal-aging SAMR1 mice and, if so, how the aging process affects the normal and disturbed cortical network activity. For this, we measured and compared several parameters of the SO across different cortical areas in anesthetized SAMP8 and SAMR1 mice at 5 and 7 months old. This phenomenological description of SAMP8 mice may give insights into the underlying cellular and synaptic alterations in AD patients and may guide future therapeutical strategies.

## Materials and Methods

### Animals

The SAM mouse was originally generated from AKR/J mice at the University of Kyoto by Dr. Takeda (Takeda, 1999). The mice included in this study were bred at the University of Barcelona and kindly provided by Dr. Pallas (Pallas et al., 2008). Five-month-old (SAMR1 *n* = 3; SAMP8 *n* = 7) and 7-month-old (SAMR1 *n* = 8; SAMP8 *n* = 9) adult female mice were included in the study of cortical electrophysiology. Mice were cared for and treated in accordance with Spanish regulatory laws (BOE 256; 25-10-1990), which comply with the European Union guidelines on protection of vertebrates used for experimentation (Strasbourg 3/18/1986). All experiments were approved by the Ethics Committee at the Hospital Clinic (Barcelona, Spain). Mice were kept under standard conditions (room temperature 23 ± 1°C, 12:12-h light-dark cycle, lights on at 08:00 a.m.), with food (A04, Harlan, Spain) and water available *ad libitum* throughout the study.

### *In Vivo* Extracellular Recordings

Anesthesia was induced by means of intraperitoneal injection of ketamine (100 mg/kg) and medetomidine (1.3 mg/kg). Atropine (0.3 mg/kg) and methylprednisolone (30 mg/kg) were administered subcutaneously to prevent respiratory secretions and inflammation, respectively. A tracheotomy was performed to increase stability during the recordings. After this procedure, the mouse was placed in a stereotaxic frame, and air enriched with oxygen was delivered to the mouse through a thin silicon tube placed at 0.5–1 cm from the tracheal cannula. Continuous infusion of ketamine (40 mg · kg^−1^ · h^−1^) was delivered subcutaneously with a pump to maintain a constant level of anesthesia (Ruiz-Mejias et al., 2011). Body temperature was maintained at 37–38°C throughout the experiment. Following Paxinos and Franklin (2008), bilateral craniotomies were made at four sites in each mouse: AP 2.3 mm from bregma, L 0.4 mm (prelimbic cortex (PrL) or medial prefrontal cortex); AP 0.5 mm, L 1.5 mm (primary motor cortex, M1); AP −1.5 mm, L 2.5 mm (primary somatosensory cortex, S1); and AP −2.5 mm, L 1.5 mm (primary visual cortex, V1). Cortical recordings were obtained from deep layers (1.1–1.2 mm deep in PrL, 0.9–1.0 mm in M1, 0.8–0.9 mm in S1 and 0.8–0.9 mm in V1) by means of 1–2 MΩ tungsten microelectrodes (tip size less than 10 μm) insulated with a plastic coating except for the tip (FHC, Bowdoin, ME, USA). Spontaneous local field potential (LFP) recordings were obtained simultaneously and bilaterally from each cortical area. LFP recordings provide information about the local neuronal population—within 250 μm according to Katzner et al. (2009)—including synaptic potentials and, when high-pass-filtered, the multiunit activity (MUA). The recording of multichannel LFPs from different areas during spontaneous activity therefore informs about the state of different neuronal ensembles, their firing rates (FRs), rhythmic activity, synchronization in different frequency bands and wave propagation. All these parameters allow a detailed comparison between SAMR1 and SAMP8 and an estimation of properties such as excitability or excitatory/inhibitory balance. In each animal, the four cortical areas were recorded in a counterbalanced order to avoid any influence of anesthesia levels, even though a constant level of anesthesia was maintained throughout the experiment. Arrays of 16 aligned electrodes separated by 100 μm (Neuronexus) were used to record activity propagation. The array was placed at 0.9–1.2 mm from the cortical surface and parallel to the midline in PrL, M1 and S1 cortices. The signal was amplified with a multichannel system (Multi Channel Systems), digitized at 20 kHz with a CED acquisition board and acquired with Spike 2 software (Cambridge Electronic Design) unfiltered.

### Data Analysis

To assess cortical network activity in SAMP8 and SAMR1 mice, we analyzed and compared several parameters of the SO: its mean frequency, Up and Down state duration, speed of Up state propagation, the coefficient of variation (CV) of the Up-Down state period, the population FR and the non-linear sample entropy (SampEn). Detection of Up and Down states from the recorded signals was based on three main fingerprints of the Up states: the slow LFP deflection, the gamma rhythm and the neuronal firing. These three features are reflected in three different time series: (1) the SO envelope; (2) the envelope of the variance of the gamma-filtered LFP (Mukovski et al., 2007); and (3) the estimation of MUA (Reig et al., 2010; Sanchez-Vives et al., 2010). From each LFP we obtained a highly processed time series as a linear combination of these three features. The contribution of each one was weighted by principal component analysis (PCA). As the three signals correspond to three different frequency bands, this method is very robust against colored noise or band-limited electrode malfunction. Up and Down states were singled out by setting a threshold in this highly processed time series. Parameters of the SO were computed from bilateral recordings in the four recorded areas. After Up and Down state detection, mean Up and Down durations were obtained. The frequency of the SO was the inverse of the duration of the whole Up-Down cycle. The CV of the frequency of the SO was the ratio between the standard deviation and its mean frequency.

For the estimation of propagation speed we recorded SO with a 16-channel array with electrodes separated by 100 μm. Up states detected in different channels were grouped in waves using a recursive algorithm. For a set of Up states to be a wave they had to appear once in each channel within a certain time window, adjusted at each iteration. Subsequently, small waves were recollected in full waves (covering a wider area) and waves involving less than 10 channels were rejected. The average of the onset times of the same wave was taken as reference to compute the array of relative time lags. In some cases, highly noisy channels were not included in the analysis. However, in all the experiments we used at least 10 electrodes. We explored the possible existence of different patterns of activity propagation by clustering the arrays of relative time lags in five groups using k-means. For each group of waves and for each electrode, we carried out the average time lag and its standard error mean (SEM). For each recording and for each group the speed of wave propagation was computed by dividing the distance between electrodes showing maximum and minimum average time lags by their time lag difference. For each recording, the speed of wave propagation was the mean of the five groups, weighted by the proportion of Up states contained in each of them.

The FR was based on the MUA signal. MUA was estimated from the extracellular recordings as the power change in the Fourier components at frequencies between 200 Hz and 1500 Hz in 5 ms windows (Reig et al., 2010; Sanchez-Vives et al., 2010). We assumed that the spectrum at this band provides a good estimate of the population FR, because Fourier components at high frequencies have densities proportional to the spiking activity of the involved neurons (Mattia and Del Giudice, 2002). To obtain MUA time series (logMUA), MUA values were logarithmically scaled in order to balance the large fluctuations of the nearby spikes. The absolute values of FR during Up and Down states were computed as the mean of logMUA values during homologous periods. The relative FR was the maximum value of the logMUA averaged waveform following the down-to-up transition, after normalized to be zero during the Down states. The complexity of the logMUA time series was obtained by computing Sample Entropy (SampEnt). We calculated SampEnt in each single Up and Down state, and then averaged the values from homologous periods. SampEn (*m, r, N*) is the negative natural logarithm of the conditional probability that two sequences of length *N*, similar for *m* points, remain similar, that is, within a tolerance *r*, at the next point, where self-matches are not included in calculating the probability (Richman and Moorman, 2000). Following previous studies (Sokunbi et al., 2013), we chose *m* = 2 and *r* = 0.25.

To analyze the fast components of the SO, we carried out Welch’s power spectrum density analysis during Up and Down states, with 50% overlapped windows of 5,000 samples. In order to highlight and compare peaks of neuronal synchronization over the intrinsic exponential decay of the signal, we computed the Power Excess, defined as the power ratio between Power and the “*1/f*” decay.

### Statistical Analysis

Comparisons between groups and ages were performed separately for each cortical area with the Student’s *t*-test for independent samples. Throughout the text comparisons between groups pooling all the cortical areas are provided, and are performed also with the Student’s *t*-test for independent samples. Unless otherwise stated, data are displayed as mean ± SE in all error bars in plots. All the analyses were implemented in MATLAB (The MathWorks, Natick, MA, USA).

## Results

**Figure 1:**
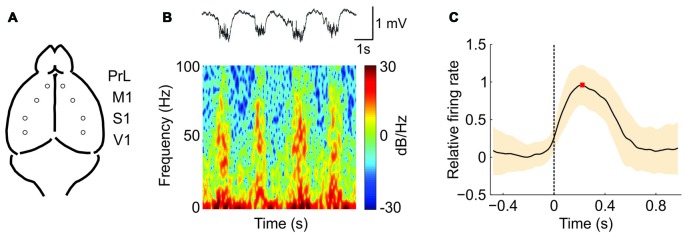
**Oscillatory activity in the PrL (prefrontal) cortex of a senescence accelerated mouse resistance 1 (SAMR1; control) mouse under ketamine anesthesia. (A)** Spontaneous extracellular activity in deep layers was recorded simultaneously in both hemispheres for each cortical area (prelimbic, PrL; primary motor, M1; primary somatosensory, S1; and primary visual, V1). **(B)** *Top*: raw local field potential (LFP) from the right PrL cortex of a SAMR1 mouse showing the alternating Up (active) and Down (silent) states. *Bottom*: spectrogram showing the presence of high frequency rhythms, mainly during the Up states. **(C)** Waveform average of the mean multiunit activity (MUA) signal aligned at the Down-to-Up state transition (*dashed line at time 0*). This average is used for the calculation of the maximum (*red square*) firing rate (FR; *see the “Materials and Methods” Section for further details*). The shade corresponds to the SD.

We recorded the extracellular spontaneous rhythmic activity generated in different regions (Figure 1A) of the SAMR1 and SAMP8 mouse neocortex (PrL, M1, S1 and V1) by means of tungsten electrodes placed in deep cortical layers. Recordings were obtained under deep anesthesia at 5 and 7 months old. Under these conditions, both SO (≤1 Hz) and fast rhythms (beta-gamma) were generated in the thalamocortical network (Steriade et al., 1993, 1996b; Ruiz-Mejias et al., 2011). The SO consisted of periods of neuronal firing, or Up states, interspersed with rather silent periods, or Down states (Figure 1B, *top*). As has been previously described (Steriade et al., 1996a,b; Ruiz-Mejias et al., 2011), we observed that during Up states, high frequency fluctuations in the beta and gamma range were generated (Figure 1B, *bottom*). Different parameters of the oscillatory activity were quantified (Sanchez-Vives et al., 2010; Ruiz-Mejias et al., 2011) and compared between control (SAMR1) and SAMP8 mice: the frequency of the SO, Up and Down state duration, speed of Up state propagation, the CV of the Up-Down state cycle and the population FR (Figure 1C). Non-linear sample entropy (SampEn) was also calculated in order to measure the complexity of the LFP dynamics during SO (Mizuno et al., 2010; Sokunbi et al., 2013). LFP recordings were obtained bilaterally but, since no significant differences were found between hemispheres for the same cortical area in any of the analyzed parameters and groups, data from both hemispheres for each cortical area are presented averaged in every animal.

### Parameters of the Slow Oscillation in Anesthetized SAMP8 Mice

**Figure 2:**
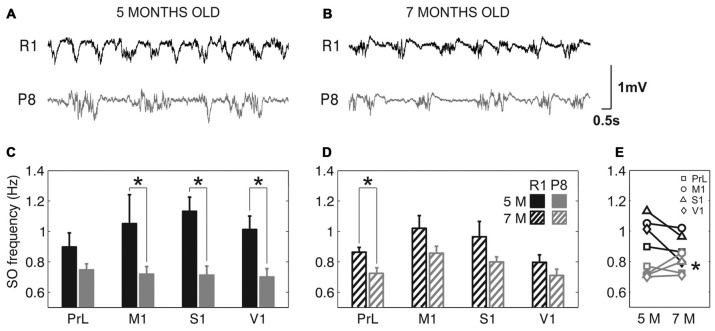
**Slow oscillation (SO) frequency in SAMR1 and SAM prone 8 (SAMP8) mice. (A,B)** Raw LFP recordings from right M1 cortex at 5 and 7 months old, respectively, in SAMR1 (*black*) and SAMP8 mice (*gray*). **(C,D)** Population data comparing the SO frequency between SAMR1 (*R1*) and SAMP8 (*P8*) mice at 5 and 7 months of age, respectively, in the four cortical areas. **(E)** Population data showing the evolution of SO frequency in the transition from 5 months to 7 months old, in the four cortical areas in both groups. Bars and symbols depict the mean, error bars are SE. *p < 0.05 SAMP8 vs. SAMR1 (*control*) in **(C,D)**, and 5 vs. 7 months of age for each cortical area and group in **(E)**. 5 M (*5 months old*), 7 M (*7 months old*). Prelimbic cortex (PrL), primary motor cortex (M1), primary somatosensory cortex (S1), primary visual cortex (V1).

**Figure 3:**
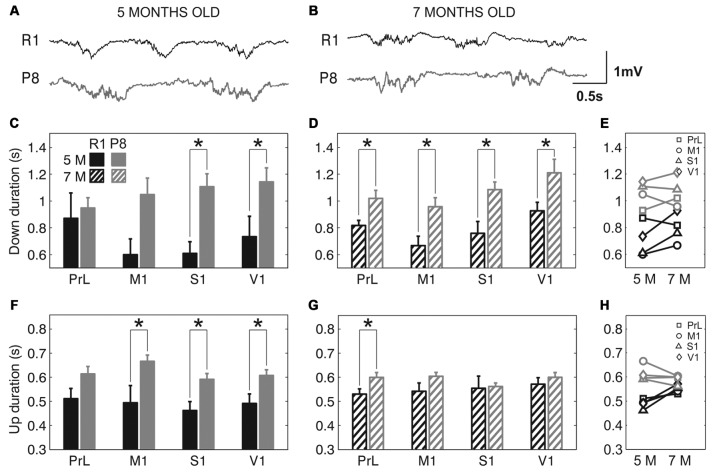
**Up and Down state duration in SAMR1 and SAMP8 mice. (A,B)** Expanded raw LFP recordings from right M1 cortex at 5 and 7 months old, respectively, in SAMR1 (*black*) and SAMP8 mice (*gray*). **(C,D)** Population data comparing Down state duration between SAMR1 (*R1*) and SAMP8 (*P8*) mice at 5 and 7 months of age, respectively, in the four cortical areas. **(F,G)** Same as above, comparing Up state duration. **(E,H)** Population data showing the evolution of Down and Up state duration, respectively, in the transition from 5 months to 7 months old in the four cortical areas in both groups. Bars and symbols depict the mean, error bars are SE. *p < 0.05 SAMP8 vs. SAMR1 (*control*) in **(C,D,F,G)**, and 5 vs. 7 months of age for each cortical area and group in **(E,H)**. Abbreviations as in Figure 2.

The frequency of the SO was reduced in SAMP8 animals compared to control ones in the four studied cortical areas, as shown in representative raw LFP traces obtained from M1 at 5 and 7 months old (Figures 2A,B, respectively). The average frequency of the SO when all the cortical areas were pooled together was also reduced in SAMP8 with respect to SAMR1 mice at both 5 (Figure 2C; 0.72 ± 0.02 vs. 1.02 ± 0.03 Hz, *p* < 0.001) and 7 months of age (Figure 2D; 0.77 ± 0.01 vs. 0.91 ± 0.02 Hz, *p* < 0.001), the differences between groups being larger in primary cortical areas (M1, S1 and V1) at 5 months old. Expanded raw LFP traces (Figures 3A,B) illustrate that the reduced frequency of the SO in the SAMP8 group was mainly driven by an increase in the Down state duration with respect to control mice (Figures 3C,D). Even though when pooling all the areas together both Up and Down state mean durations were longer in SAMP8 than in SAMR1 mice at 5 months old (Figures 3C,F; Down: 1.1 ± 0.04 vs. 0.71 ± 0.05 s, *p* < 0.001; and Up: 0.61 ± 0.01 vs. 0.49 ± 0.01 s, *p* < 0.001), the difference with respect to control mice was of greater magnitude for Down state duration (an almost two-fold increase across all primary cortical areas), and persisted and was emphasized at 7 months, being statistically significant in all the cortical areas separately (Figures 3D,E). Conversely, Up state duration tended to converge between groups at 7 months (Figures 3G,H), reducing the SO frequency of the control group (Figure 2E). Therefore, the elongation of the Down state in SAMP8 is a consistent difference with respect to control mice at the two studied ages.

**Figure 4:**
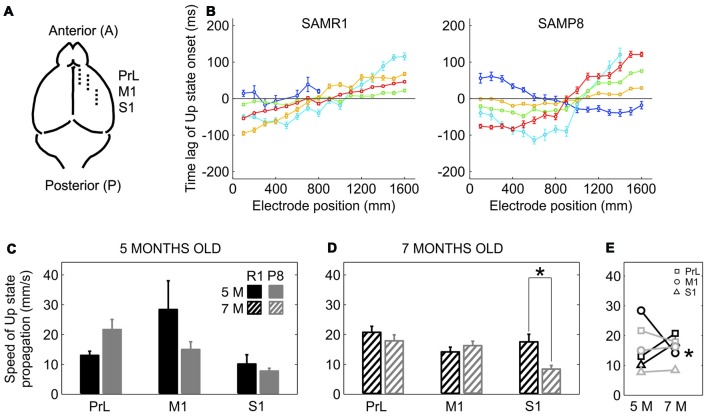
**Speed of propagation of Up state onsets in SAMR1 and SAMP8 mice. (A)** Location and position of the 16 channel recording array (*discontinuous line*) in the PrL, M1 and S1 cortices. **(B)** Average time lags of Up state onsets recorded in the M1 area of a 5-month-old R1 (*left*) and P8 mice (*right*). Time lags were grouped in five different pools with similar patterns of activity propagation (*each with different color*) obtained with a principal component analysis (PCA; *see the “Materials and Methods” Section for details*). **(C,D)** Population data comparing the speed of Up state propagation between SAMR1 (*black*) and SAMP8 (*gray*) mice at 5 and 7 months old, respectively, in the three cortical areas. **(E)** Population data showing the evolution of Up state propagation in the transition from 5 months to 7 months old in the three cortical areas in both groups. Bars and symbols depict the mean, error bars are SE. *p < 0.05 SAMP8 vs. SAMR1 (*control*) in **(C,D)**, and 5 vs. 7 months of age for each cortical area and group in **(E)**. Other abbreviations as in Figure 2.

The elongation of the Down state inevitably went along with a lower frequency of Up state generation in SAMP8 animals. We next examined whether this lower frequency of Up state generation was also accompanied by a lower speed of Up state propagation with respect to control mice. Up states can originate locally from anywhere in the neocortex, but most of them propagate as traveling waves across the cortical tissue (Massimini et al., 2004; Luczak et al., 2007; Ruiz-Mejias et al., 2011; Stroh et al., 2013). Such traveling waves originate preferentially in the prefrontal region and propagate in the anteroposterior direction (Massimini et al., 2004). We evaluated wave propagation with an array of 16 electrodes placed parallel to the midline, in PrL, M1 and S1 cortices (Figure 4A). Coincident with previous reports (Ruiz-Mejias et al., 2011), the vast majority of waves traveled in the anteroposterior direction in both control and SAMP8 mice (Figure 4B). Although the speed of Up state propagation when pooling all the cortical areas together tended to be lower in SAMP8 than in control mice at both 5 (Figure 4C; 15.55 ± 2 vs. 17. 2 ± 4.1 mm/s, *p* = 0.68) and 7 months of age (Figure 4D; 14. 51 ± 1.3 vs. 17.5 ± 1.3 mm/s, *p* = 0.1), differences between groups when considering each cortical area separately were only significant in the S1 cortex of 7 month-old animals (Figure 4D). The speed of wave propagation hardly varied with age in SAMP8 animals, but in control ones showed either a tendency to increase or decrease in the transition from 5 months to 7 months of age, depending on the cortical area (Figure 4E).

**Figure 5:**
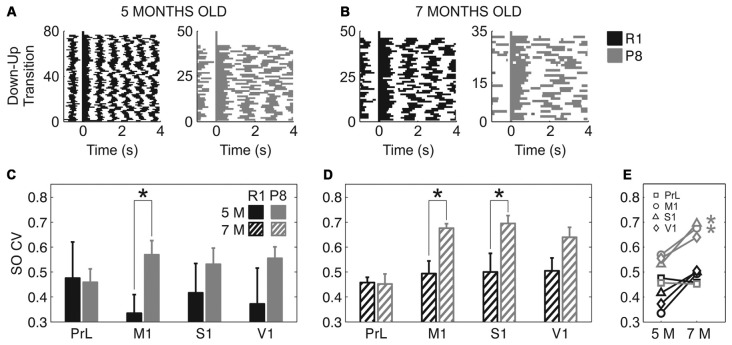
**Coefficient of variation (CV) of the SO in SAMR1 and SAMP8 mice. (A,B)** Representative raster plots of aligned Up states (detected during 1-min recordings) in M1 cortex at 5 and 7 months old, respectively, in a single SAMR1 (*black*) and SAMP8 (*gray*) mice. Decreased SO frequency and regularity in SAMP8 mice are clearly illustrated in these examples. **(C,D)** Population data comparing the CV of the SO between SAMR1 and SAMP8 mice at 5 and 7 months of age, respectively, in the four cortical areas. **(E)** Population data showing the evolution of the CV of the SO in the transition from 5 months to 7 months old in the four cortical areas in both groups. Bars and symbols depict the mean, error bars are SE. *p < 0.05 SAMP8 vs. SAMR1 (*control*) in **(C,D)**, and 5 vs. 7 months of age for each cortical area and group in **(E)**. Abbreviations as in Figure 2.

In addition to a reduced frequency of the SO and a trend towards slower speed of wave propagation, SAMP8 mice also presented a more irregular SO cycle than did SAMR1 mice (Figure 5). This irregularity can be observed in single cases if we represent consecutive Up states aligned with respect to their Down-to-Up transition in raster plots (Figures 5A,B), which provide information about the duration of the Up and Down states and their variability. Rasters corresponding to the SAMP8 mice evidenced the elongation of Up and Down states and a larger irregularity in their time occurrence. These observations could be validated at the population level. The mean CV of the SO when pooling all the areas together was significantly increased in SAMP8 with respect to control mice at both 5 (Figure 5C; 0.53 ± 0.02 vs. 0.4 ± 0.05, *p* < 0.05) and 7 months of age (Figure 5D; 0.61 ± 0.02 vs. 0.48 ± 0.02, *p* < 0.001). This increase in cycle variability was driven by the primary cortices, while it remained rather constant in PrL cortex, which displayed the maximal regularity among the studied areas and showed no differences between and within groups at any of the studied ages. Conversely, the CV of the SO in primary cortical areas was higher in SAMP8 than in control animals, and increased in the transition from 5 months to 7 months of age in both groups, although this increase was of higher magnitude in SAMP8 mice (Figure 5E). Interestingly, this observation would be consistent with the prefrontal area being the most common origin of a new wave (Massimini et al., 2004) and also the most regular one, as it has been described in the mouse (Ruiz-Mejias et al., 2011). As was the case with SO frequency and Up and Down state durations, the CV of the SO in SAMR1 approached at 7 months the CV in SAMP8 at 5 months old, perhaps suggesting that SAMR1 approach the phenotype of the senescence-accelerated SAMP8 with aging.

**Figure 6:**
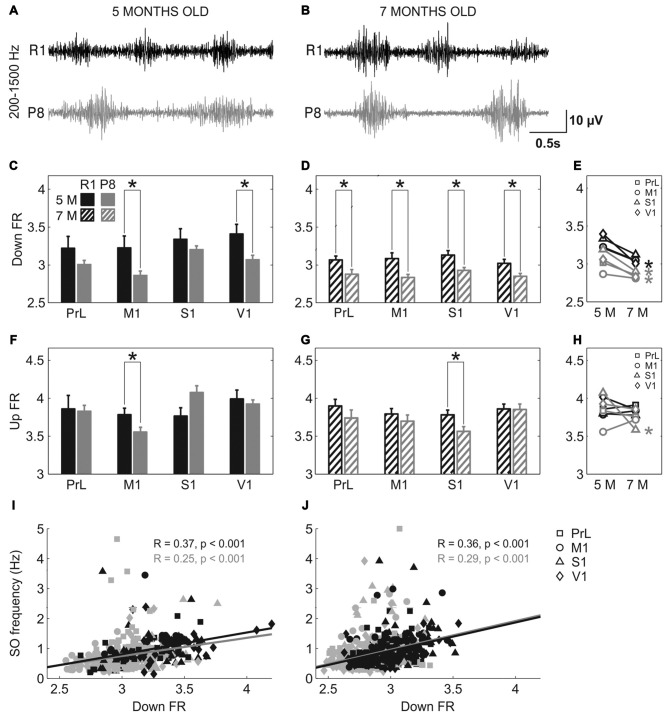
**Population FR in SAMR1 and SAMP8 mice. (A,B)** Filtered LFP recordings (200–1500 Hz) from right M1 at 5 and 7 months old, respectively, in SAMR1 (*black*) and SAMP8 mice (*gray*). **(C,D)** Population data comparing the FR during Down states between SAMR1 and SAMP8 mice at 5 and 7 months of age, respectively. **(F,G)** Same as above, comparing FR during Up states. **(E,H)** Population data showing the evolution of Down and Up state FR, respectively, in the transition from 5 months to 7 months old in the four cortical areas in both groups. **(I,J)** Pearson’s correlation between the FR during Down states and the frequency of the SO at 5 and 7 months of age, respectively, in the four cortical areas. Bars and symbols depict the mean, error bars are SE. *p < 0.05 SAMP8 vs. SAMR1 (*control*) in **(C,D,F,G)**, and 5 vs. 7 months of age for each cortical area and group in **(E,H)**. Abbreviations as in Figure 2.

Population FR during Up and Down states was estimated by analyzing the power spectrum density of the SO between 200 and 1500 Hz (Mattia and Del Giudice, 2002; Figure 6). The high-pass filtered LFP that corresponds to the MUA is represented in Figure 6A (5 months old) and Figure 6B (7 months old). Mean FR during the Down states when pooling all the areas together was reduced in SAMP8 with respect to the control group at 5 (Figure 6C; 3.03 ± 0.03 vs.3.3 ± 0.06, *p* < 0.001) and 7 months of age (Figure 6D; 2.85 ± 0.02 vs. 3.05 ± 0.03, *p* < 0.001), the differences between groups being larger in 7-month-old animals. Smaller differences were observed in the FR during the Up state (Figures 6F,G), although when pooling all the areas together the FR was reduced in SAMP8 with respect to the control group at 7 months of age (Figure 6G; 3.72 ± 0.04 vs. 3.84 ± 0.03, *p* < 0.05). However, these differences were less consistent than those occurring in the Down state. Furthermore, the FR during the Down state decreased in the transition from 5 months to 7 months of age in both groups (Figure 6E), while no substantial change occurred in the case of Up states (Figure 6H). This reduced FR during the Down states in the SAMP8 neocortex could be related to its difficulty in generating a new Up state, thus causing the elongation of the Down state and consequently decreasing the SO frequency (Compte et al., 2003). Indeed, Down state FR was positively correlated with the frequency of the SO at the two ages in both control and SAMP8 mice (Figures 6I,J).

**Figure 7:**
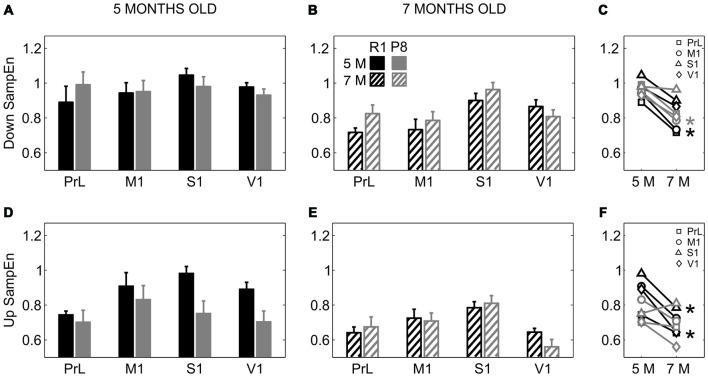
**Complexity of LFP dynamics during SO in SAMR1 and SAMP8 mice. (A,B)** Population data comparing sample entropy (SampEn) as a measure of complexity during Down states at 5 and 7 months of age, respectively, in SAMR1 (*black*) and SAMP8 (*gray*) mice. **(D,E)** Same as above, comparing SampEn during Up states. **(C,F)** Population data showing the evolution of Down and Up state SampEn, respectively, in the transition from 5 months to 7 months old in the four cortical areas in both groups. Bars and symbols depict the mean, error bars are SE. *p < 0.05 SAMP8 vs. SAMR1 (*control*) in **(A,B,D,E)**, and 5 vs. 7 months of age for each cortical area and group in **(C,F)**. Abbreviations as in Figure 2.

To investigate whether SAMP8 mice presented altered LFP dynamics complexity with respect to control mice, we computed the SampEn during Up and Down states separately. SampEn is related to the number of times the patterns in a signal are repeated, hence providing a measure of its randomness and predictability, with lower values of SampEn indicating lower complexity of the signal or system (Sokunbi et al., 2013). While no differences in SampEn were found between groups during the Down states (Figures 7A,B), SampEn during the Up states was reduced in 5-month-old SAMP8 mice compared to control ones when pooling all the cortical areas together (Figure 7D; 0.75 ± 0.03 vs. 0.88 ± 0.03, *p* < 0.05), while it presented no differences at 7 months old (Figure 7E). Up and Down state SampEn clearly tended to decrease with age in both groups of animals, representing a decreased complexity with age, especially that during the Up states in the control group, again converging with SAMP8 animals and canceling the differences between groups observed at 5 months (Figures 7C,F).

### Fast Components of SAMP8 Cortical Slow Oscillations

Finally, we studied the high frequency content of the LFP during Up and Down states in SAMP8 and control mice. We were especially interested in the synchronization of the cortical activity at high frequency bands (beta-gamma), as this synchronization has been widely linked to cognitive and perceptual processes (Wang, 2010; Siegel et al., 2012). This analysis showed that mean power spectra of SAMP8 differed from those of control mice at both ages and in all the studied cortical areas.

**Figure 8:**
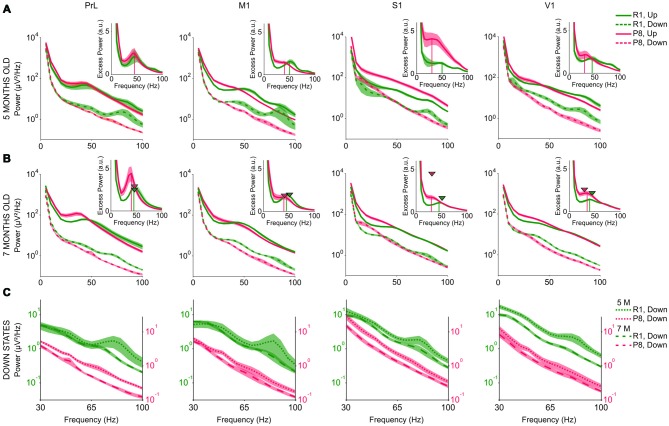
**High frequency content of the SO in SAMR1 (*green*) and SAMP8 mice (*red*). (A,B)** Mean raw power spectra at 5 and 7 months of age, respectively, in PrL, M1, S1 and V1 cortices (*from left to right*), during Up (*solid lines*) and Down states (*dashed lines*). *Insets* show excess power during the Up states, defined as the ratio between the mean power during Up states and the fit of the “*1/f*” decay of the power spectrum. Vertical lines in the insets indicate the power peak for each group. Arrow heads in the insets of **(B)** indicate the frequency and amplitude of the power peak at 5 months old. **(C)** Mean power during the Down states at 5 (*dotted line*) and 7 months old (*dashed line*) in SAMR1 (*green*) and SAMP8 (*red*) groups. Shadow is the SE. a.u. (arbitrary units). Other abbreviations as in Figure 2.

During Up states (Figures 8A,B, *solid lines*), SAMP8 mice presented an overall increase of power in the lower range of the beta-gamma band compared to control mice. This increase was also accompanied by a trend towards reduced power in the upper range, thus giving the impression that the spectral profile of SAMP8 mice was shifted towards lower frequencies. In fact, the power peak in the excess power of the Up states appeared displaced towards lower frequencies in SAMP8 compared to control mice at both studied ages and all the four cortical areas (Figures 8A,B, *vertical lines in the insets*). Modulations in the beta-gamma band could be observed in both groups of animals in PrL cortex but, unlike control mice, the synchronization of the cortical activity in the beta-gamma range did not form a distinguishable modulation in the primary cortical areas of the SAMP8 group, suggesting reduced beta-gamma rhythmicity in these animals. Furthermore, control animals showed another modulation in the upper gamma range during both Up and Down states that was highly attenuated in the transition from 5 months to 7 months old, a modulation that was not present in the SAMP8 group at any of the studied ages. As mice got older, high frequencies during the Up states displayed different behaviors in PrL and primary cortices depending on the group. The power peak in PrL increased in both control and SAMP8 animals, and shifted to lower frequencies in the second ones (Figure 8B, *arrow heads vs. vertical lines in the insets*). Conversely, the displacement to lower frequencies occurred in the primary cortical areas for the control group, while the peak in these areas tended to remain stable in SAMP8 mice. Special attention to the accentuated overall larger power in the S1 area of 5-month-old SAMP8 mice is warranted (Figure 8A). This large difference with respect to control mice disappeared in the transition from 5 months to 7 months old, and was also accompanied by a significant decrease in the Up state FR (Figure 6H).

High frequency content during Down states was also altered in the SAMP8 group. Similar to the changes observed in FR, high frequencies during the Down state presented lower power in SAMP8 than in SAMR1 mice, in all the cortical areas (Figures 8A,B, *dashed lines*). Furthermore, SAMR1 and SAMP8 animals exhibited significant reductions of power in the transition from 5 months to 7 months of age, even though the power of high frequencies during the Down state was always lower in SAMP8 than in control mice (Figure 8C).

## Discussion

In this study we compared the emergent cortical network activity in senescence-accelerated SAMP8 mice with normal-aging SAMR1 mice under ketamine anesthesia at two different ages. Under these conditions, Up and Down states were spontaneously generated by the cortical network, and different parameters of the slow oscillatory activity and the embedded fast (beta-gamma) rhythms were quantified and compared between SAMP8 mice and its control, SAMR1. Our experiments show that SAMP8 mice present disturbances in the cortical network activity when compared with SAMR1 mice, and that the electrophysiological phenotype of SAMR1 at 7 months old tends to approach that of SAMP8 at 5 months old.

SO recorded in the cerebral cortex of SAMP8 mice were slower than those of control mice. Although both Up and Down states tended to be longer in the SAMP8 strain, Down state elongation was the most robust and consistent difference across ages, a finding coincident with SO alterations reported in a mouse model of tauopathy (Menkes-Caspi et al., 2015). This reduction in the SO frequency in SAMP8 animals was accompanied by a decrease in its regularity across primary cortical areas (motor, somatosensory and visual) although, interestingly, the cycle variability of the PrL cortex presented no differences between groups, showing a tendency to be the area with the maximal regularity in the two groups at both studied ages. This result is consistent with previous observations in anesthetized mice (Ruiz-Mejias et al., 2011) and coherent with the view of the prefrontal cortex (the equivalent to the mouse PrL cortex) as the most common initiator of new waves (Massimini et al., 2004). Indeed, the preferred direction of wave propagation was the anteroposterior for both SAMP8 and SAMR1 mice, suggesting that this property of the prefrontal cortex is preserved in SAMP8 animals.

SAMP8 also presented reduced FR compared with control mice, and this difference between groups was emphasized at 7 months of age, being more prominent during the Down sates. This decrease in high frequency fluctuations during the Down state might be contributing to the difficulty of the network to generate a new Up state (Compte et al., 2003), thus producing Down state elongation and the subsequent reduction in the SO frequency. In support of this idea, the FR during the Down states was highly correlated with the frequency of the SO in all the cortical areas of SAMR1 and SAMP8 animals. Furthermore, Down state FR decreased in the transition from 5 months to 7 months old in both SAMP8 and SAMR1 mice, and this was accompanied by a reduction in the SO frequency, especially in the control mice, as in the SAMP8 group this frequency was already very low at 5 months of age. The magnitude of noise during the Down states has also been related to the regularity of the cycle (Sancristobal et al., 2016), demonstrating that the relationship between these two parameters follows a U-shape function in which low and high levels of noise during the Down state result in higher variability, and that there is an intermediate optimal level of noise for which the regularity is the highest. As the animals got older, FR and entropy during the Down states decreased in SAMP8 and SAMR1 animals, and this was accompanied by a subsequent increase in the cycle variability of the SO in the primary cortical areas, but not in the PrL cortex. This may suggest that this optimal background noise admits a wider range of FR values in prefrontal than in primary cortices before it causes a rise in the variability of the cycle.

Although the vast majority of differences between the groups were consistent across both studied ages, the phenotype of control mice at 7 months old tended to converge with that of SAMP8 mice at 5 months old. Little is known about the underlying genetics that predispose SAMP8 mice to an accelerated senescence. Genetic expression studies have identified some specific alterations differentially expressed in SAMP8 and SAMR1 mice (Carter et al., 2005; Pallàs, 2012). For example, a deficiency of MEF2C in SAMP8, a gene related to the regulation of cortical synapses, could be the cause of some of the observed functional differences. However, it is still unclear whether SAMP8 mice display physiological–albeit accelerated–aging or, conversely, pathological aging with mechanisms different from those acting in the normal aging process. The fact that 7-month-old SAMR1 mice tended to exhibit some properties similar to those of SAMP8 at 5 months old in several of the parameters studied here suggests that the differences between them could be the result of different life phase equivalences, instead of separate aging mechanisms. This is well reflected in the evolution of the SO frequency, the Up state duration, the regularity of the cycle and the Down state FR, and also in the high frequencies during the Up state. As SAMR1 got older, the power peak of beta-gamma synchronization was displaced towards lower frequencies, approaching the frequency of the peak in SAMP8 animals. Interestingly, this happened in all the cortices except in the PrL cortex and, conversely, the opposite pattern could be observed in SAMP8 mice, suggesting that gamma synchronization disturbances are belatedly expressed in the PrL area.

One of the principal hallmarks of electroencephalography (EEG) abnormalities in AD patients is a shift of the power spectrum to lower frequencies (Jeong, 2004; Lizio et al., 2011; Roh et al., 2011) and this—among other alterations—correlates with the severity of the disease (Kowalski et al., 2001). Here we found that the synchronization peak in the gamma range during the Up states occurred at lower frequencies in SAMP8 than in control mice, and that the mean power spectrum between 0 Hz and 100 Hz presented an overall increase in its lower range together with a decrease in the upper range. These findings are substantially reminiscent to what has been described in human patients with AD, and add evidence in support of the suitability of the SAMP8 mouse as a model of AD, given the few reports using mouse models that find EEG alterations consistent with human observations (Sabbagh et al., 2013). There is substantial evidence that high frequency synchronization in the beta-gamma range represents the network’s correlate of attentive and cognitive processes (Wang, 2010; Siegel et al., 2012), and that the inhibitory neurons play a critical role in the generation of these rhythms (Tamas et al., 2000; Hasenstaub et al., 2005; Compte et al., 2008; Rubio et al., 2012; Verret et al., 2012). The results obtained here demonstrate that spectral profile changes that take place with time also occur in control animals, suggesting that alterations in GABAergic inhibition might be central to the aging process in the SAM strains. However, altered high frequency synchronization in the PrL cortex was only observed in SAMP8 mice. As previously stated, this could point out to a greater resistance of this area to the aging process or, alternatively, it could indicate that beta-gamma alterations in the PrL cortex are specific of the SAMP8 group and may partially explain the cognitive deficits reported in these animals (Flood and Morley, 1998).

An increase in cortical excitability has been extensively described in mouse models of AD, which have a marked tendency to suffer epileptic seizures (Palop et al., 2007; Palop and Mucke, 2009; Gurevicius et al., 2013; Siskova et al., 2014; Siwek et al., 2015), and also in human patients with AD (Amatniek et al., 2006; Hommet et al., 2008; Khedr et al., 2011; Larner and Marson, 2011; Born, 2015). Nevertheless, the elongation of the Down state, the reduced frequency of the SO, the tendency towards slower speed of propagation and the diminished FR reported in the present study suggest rather a reduced cortical excitability in SAMP8 animals relative to controls. The discrepancy could be explained by the fact that the vast majority of studies reporting higher cortical excitability in AD have been conducted in human patients and transgenic mouse models of AD that present large β-amyloid deposition, which has been directly related to neuronal hyperexcitability (Busche et al., 2008, 2015). However, there is no plaque formation in the cerebral cortex of 5- and 7-month-old SAMP8 mice (Takeda, 2009; Cheng et al., 2014). Thus, it seems that factors other than amyloid deposits but associated to aging are involved in the network alterations reported here.

In conclusion, we have found that SAMP8 mice present disturbances in cortical network activity when compared with SAMR1 (control) mice and that the electrophysiological phenotype of control mice at 7 months of age tends to approach the phenotype of SAMP8 mice at 5 months of age. To our knowledge, this is the first systematic characterization of the cortical slow and fast rhythms in the SAMP8 model of early aging. We find that this approach is valuable to identify alterations in the cortical emergent activity of SAMP8 mice and, more generally, can provide useful insights about the cellular and synaptic transformations associated with aging and neurodegenerative diseases such as AD. The detected alterations may contribute to the SAMP8’s accelerated, progressive cognitive decline and may be related to other AD-like neurodegenerative changes previously reported in this mouse model. Despite the differences between the SAMP8 model and patients with Alzheimer’s disease, our findings highlight the electrophysiological similarities and therefore emphasize the value of the SAMP8 strain as a model for studying AD.

## Author Contributions

PC-P, MVS-V and RME designed the study. PC-P carried out the experiments, participated in the analysis and wrote the article. LP-M, MP-Z and PC-P carried out the data analysis. MVS-V supervised experiments and analysis. All authors participated in the scientific discussion and in the article writing.

## Conflict of Interest Statement

The authors declare that the research was conducted in the absence of any commercial or financial relationships that could be construed as a potential conflict of interest.
